# Effects of oleic acid on cell proliferation through an integrin-linked kinase signaling pathway in 786-O renal cell carcinoma cells

**DOI:** 10.3892/ol.2013.1160

**Published:** 2013-01-29

**Authors:** ZHENHUA LIU, YUNBEI XIAO, YEQING YUAN, XIAOWEI ZHANG, CAIPENG QIN, JING XIE, YICHANG HAO, TAO XU, XIAOFENG WANG

**Affiliations:** Department of Urology, Peking University People’s Hospital, Beijing 100044, P.R. China

**Keywords:** oleic acid, renal cell carcinoma, cell proliferation, integrin-linked kinase

## Abstract

An increased risk of renal cell carcinoma (RCC) has been linked with obesity and metabolic syndrome. However, the mechanisms by which lipid metabolic disorders affect the development of RCC remain unclear and highly controversial. Integrin-linked kinase (ILK) is a serine/threonine protein kinase involved in the regulation of tumor cell growth and angiogenesis. In the present study, the effect of free fatty acids in the promotion of RCC progression was investigated by upregulating ILK. Results of the MTT assay indicated that treatment of 786-O cells with oleic acid induced a concentration-dependent increase in cell viability. Flow cytometry analysis revealed that the effect of oleic acid on cell apoptosis was not significant. Following treatment with oleic acid, the expression of ILK, phospho-Akt and G protein-coupled receptor 40 (GPR40) was increased in 786-O cells. These effects were reversed when the expression of ILK was downregulated using specific small interfering RNA. These results indicate that free fatty acids are associated with the development of renal cell carcinoma via activation of the GPR40/ILK/Akt pathway, revealing a novel mechanism for the correlation between metabolic disturbance and renal carcinoma.

## Introduction

Renal cell carcinoma (RCC), the most common pathological pattern, accounts for 3% of adult malignancy tumors and worldwide incidence continues to increase steadily each year (1). The 5-year survival rate is 80–90% for pT1 and pT2 stage RCC; however, in patients with metastatic disease, it is only 10% (2,3). Therefore, the identification of methods to reduce risk factors and novel therapeutics is extremely important (4). Previous studies have demonstrated that patients with metabolic diseases, including diabetes mellitus and obesity, are associated with a higher risk of developing various types of cancer, including renal (5,6). Lipid metabolism disorder with high levels of serum free fatty acids (FFAs) is an important characteristic in metabolic diseases and FFAs are associated with cell development and invasion in breast and prostate cancer, indicating the role of FFAs in renal cancer (7,8).

FFAs have been revealed to stimulate intracellular signal transduction, functioning as second messengers in vascular smooth muscle and tumor cells (9,10); however, the mechanism by which FFAs affect tumor development in RCC remains unclear. Integrin-linked kinase (ILK) is a serine/threonine protein kinase, involved in the regulation of cell growth/survival, cell cycle progression, invasion and migration and tumor angiogenesis (11). ILK may activate downstream kinases, including Akt and GSK3β (12), therefore we hypothesized that the function of FFAs may be markedly associated with the ILK pathway in RCC. In the present study, the effect of the highest content of FFAs in serum, namely oleic acid, on human RCC 786-O cells was investigated *in vitro* and the mechanism by which FFAs function was determined.

## Materials and methods

### Reagents

Oleic acid and de-fatty bovine serum albumin (d-BSA) were purchased from Sigma-Aldrich (St. Louis, MO, USA). Oleic acid was supplemented with d-BSA, which functioned as a carrier to ensure sufficient dissolution (mol/mol <2). The annexin V-FITC apoptosis detection kit was purchased from Biosea Biotechnology Co. Ltd. (Beijing, China). ILK small interfering RNA (siRNA) and control non-silencing siRNA were purchased from Cell Signaling Technology Inc. (Danvers, MA, USA). Polyclonal anti-ILK, anti-Akt, anti-p-Akt ser473 and anti-G protein-coupled receptor 40 (GPR40) antibodies were purchased from Cell Signaling Technology.

### Cell culture and oleic acid treatment

Human RCC cell line, 786-O, was obtained from American Type Culture Collection (Manassas, VA, USA) and routinely cultured in RPMI-1640 medium supplemented with 10% fetal bovine serum (FBS) and 100 U/ml penicillin and streptomycin. For treatment, cells were cultured in growth medium for 24 h and then the medium was replaced with oleic acid-enriched medium at various concentrations of oleic acid (0.05, 0.1, 0.2 mmol/l). The control group received d-BSA alone at an equal concentration.

The study was approved by the Ethics Committee of Peking University People’s Hospital, Beijing, China.

### MTT assay

Cells (2×10^3^ cells/well) were seeded in 96-well microtitre plates and incubated for 24 h in 100 *μ*l culture medium. Cells in the experimental group were then treated with 0.05, 0.1 or 0.2 mmol/l oleic acid for 48 h. MTT [20 *μ*l (5 g/l)] was added to the cells which were then cultivated for a further 4 h. Following the removal of the supernatant fluid, 150 *μ*l/well DMSO was added to the cells which were agitated for 15 min. Absorbance was measured at 490 nm by an ELISA reader. Untreated 786-O cells served as controls. Each assay was repeated three times. The relative growth rate of 786-O cells was calculated using the following equation: cell viability rate (%) = (OD_oleic acid_/OD_control_) × 100.

### Apoptosis assessed by flow cytometry

The extent of apoptosis was evaluated by annexin V-FITC and flow cytometry. Cells were grown at a density of 1×10^6^ cells in 6-well culture dishes and were treated with 0.05, 0.1 or 0.2 mmol/l oleic acid for 48 h. Following treatment, cells were harvested, washed twice with pre-chilled PBS and resuspended in 1X binding buffer at a concentration of 1×10^6^ cells/ml. The solution (100 *μ*l) was mixed with 5 *μ*l annexin V-FITC and 5 *μ*l PI for 15 min, then 400 *μ*l 1X binding buffer was added. Analysis was performed using a FACStar cytofluorometer with CellQuest Pro software.

### Treatment with ILK siRNA

siRNA duplexes specific for human ILK were used to specifically knock down ILK. For the transfection procedure, cells were grown to 60% confluence and ILK and control siRNA were transfected using the Oligofectamine reagent according to the manufacturer’s instructions. Briefly, Oligofectamine reagent was incubated with serum-free medium for 10 min. Subsequently, a mixture of respective siRNA was added. Following incubation for 15 min at room temperature, the mixture was diluted with medium and added to each well. The final concentration of siRNA in each well was 100 nmol/l. Following culture for 32 h, cells were washed and resuspended in oleic acid-enriched culture medium for an additional 48 h for cell growth assays and western blot analysis.

### Western blot analysis

Cell lysates were boiled with 5X loading buffer and then fractionated by SDS-PAGE. Proteins were transferred to PVDF membrane and incubated with primary specific antibodies against GPR40, ILK, Akt and phosphor-Akt in 5% milk. Blots were washed and horseradish peroxidase-conjugated anti-mouse or anti-rabbit antibodies were applied. Blots were washed, transferred to freshly made enhanced chemiluminescence solution for 5 min and images were captured using the ImageQuant Gel imaging system.

### Statistical analysis

Data are expressed as mean ± SD for groups and analyzed using the Student’s t-test to determine differences between the oleic acid-treated and control groups. P<0.05 was considered to indicate a statistically significant difference.

## Results

### Oleic acid increases 786-O cell proliferation

**Figure 1:**
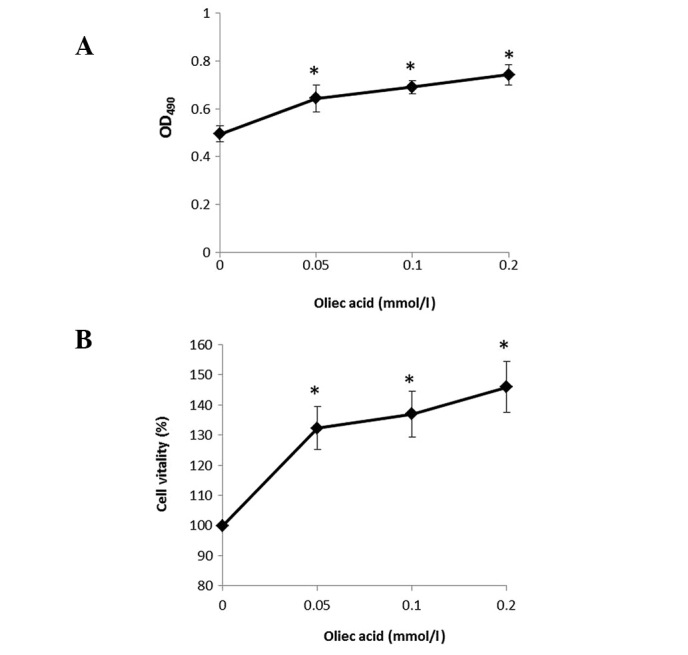
Oleic acid affected cell viability of the renal cell carcinoma (RCC) cell line, 786-O. Cells were treated with various concentrations of oleic acid for 48 h. Cell viability was measured by MTT assay. (A) Optical density and (B) relative cell viability. Data are presented as the mean ± SD. ^*^P<0.05.

To examine the function of oleic acid on the human RCC cell line, 786-O cells were cultured and treated with various concentrations of oleic acid and cell viability was detected using the MTT assay. Percentages of viable cells when cultured with oleic acid (0.05, 0.1 and 0.2 mmol/l) relative to the control-treated cells were determined at 4 h. The results indicate that oleic acid treatment induced a marked increase in the proliferation of 786-O cells in a dose-dependent manner (Fig. 1A and 1B).

### Oleic acid-delayed apoptosis of 786-O cells

**Figure 2:**
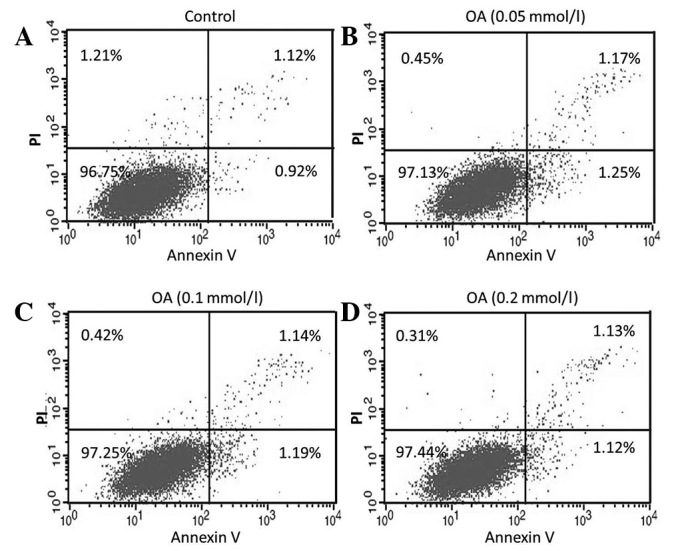
Delayed apoptosis of 786-O cells by treatment of oleic acid. Cells treated with various concentrations of oleic acid were double-stained with annexin V and PI and analyzed by flow cytometry. The gate was set to distinguish between living (bottom left), necrotic (top left), early apoptotic (bottom right) and late apoptotic (top right) cells. ^*^P<0.05; vs. the control group. OA, oleic acid; PI, propidium iodide.

To determine the effect of oleic acid on the apoptosis of 786-O cells, annexin V/PI was used. It was observed that, following treatment of 786-O cells for 48 h with oleic acid (0.05, 0.1 and 0.2 mmol/l), the total percentage of apoptotic cells was not directly associated with oleic acid concentration, decreasing from 2.42 to 2.33 and 2.25%, compared with the control cells (2.04%) (0.05, 0.1 and 0.2 mmol/l oleic acid, respectively; Fig. 2); consistent with results of the MTT assay. The results revealed that oleic acid delayed 786-O cell apoptosis in a concentration-dependent manner.

### ILK is upregulated by oleic acid treatment in 786-O cells

**Figure 3:**
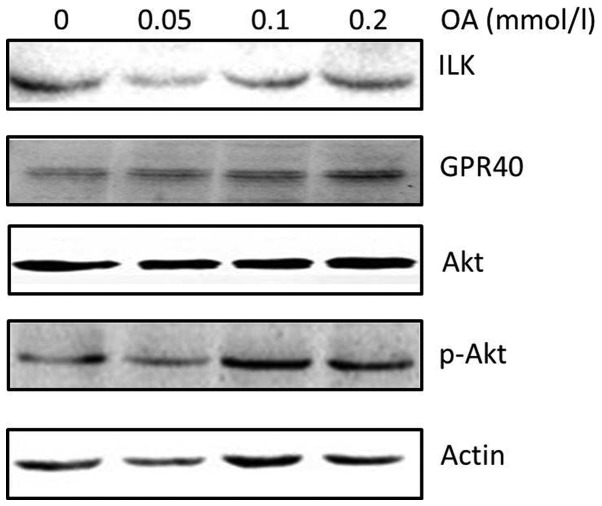
Protein expression in 786-O cells treated with various concentrations of oleic acid for 48 h. Oleic acid treatment led to upregulation of ILK and its target gene p-Akt. GPR40 was increased with the induction of apoptosis by oleic acid. Actin was used as internal control. OA, oleic acid; ILK, integrin-linked kinase; GPR40, G protein-coupled receptor 40.

Since oleic acid treatment induced 786-O cell proliferation and delayed cell apoptosis, the effect of oleic acid on specific cell proliferation regulatory molecules was analyzed. Western blot analysis revealed that oleic acid treatment induced upregulation of ILK and phospho-Akt protein, a key regulator of cell proliferation, in a dose-dependent manner (Fig. 3). To determine the mechanims by which oleic acid activates intra-cellular signaling, the expression of GPR40 protein, a specific receptor of long chain fatty acids, was determined. GRP40 protein expression was also found to be higher following oleic acid treatment in 786-O cells (Fig. 3).

### ILK siRNA reverses the effects of oleic acid on cell growth and gene expression

**Figure 4:**
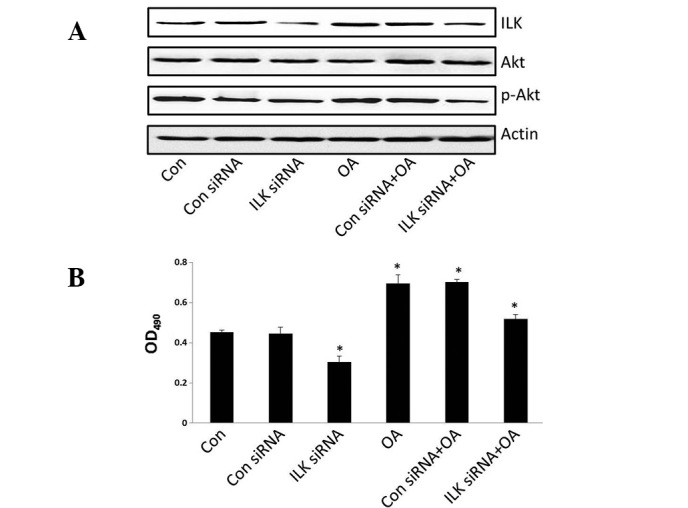
Role of ILK in mediating the effect of oleic acid on cell growth. 786-O cells were transfected with control or ILK siRNA for 32 h followed by oleic acid exposure for an additional 48 h. Viable cells were detected by MTT assay. (A) Western blot analysis of expression of specific proteins and (B) quantification. Actin used as internal control. Data are presented as mean ± SD. ^*^P<0.05; vs. the control group. OA, oleic acid; ILK, integrin-linked kinase.

To determine the role of ILK on 786-O cell proliferation, siRNA tranfection was performed to downregulate ILK expression. Cell viability was suppressed following transfection with ILK siRNA and treatment with oleic acid (Fig. 4B) and the expression of ILK and phospho-Akt was decreased (Fig. 4A). The results indicated that cell proliferation is markedly associated with the expression of ILK.

## Discussion

Previous epidemiological and animal studies have identified an association between dietary fatty acids and metabolic diseases characterized by hyperlipidemia and an elevation in circulating FFAs and have indicated that this association also correlates with enhanced cancer risk (13–16). The long chain polyunsaturated fatty acids (PUFA) in daily diets include n-3 PUFA, n-6 PUFA and others, and exhibit various roles in cancer. Previous studies have demonstrated that n-3 PUFA suppresses tumor cell proliferation and induces cell apoptosis, while n-6 PUFA promotes tumor development (17,18).

Oleic acid is an n-9 monounsaturated fatty acid, which activates G protein-coupled receptors and phosphorylates ERK1/2 to induce cancer cell proliferation in breast cancer (19). The concentration of oleic acid in normal serum is ∼0.05 mmol/l; however, a number of studies have reported that oleic acid may affect tumor cell development when its concentration is >0.025 mmol/l (20). In the present study, high concentration levels of oleic acid were used to imitate the effects of abnormal levels of FFAs on tumor growth. The results of the MTT assay indicated that specific concentrations of oleic acid stimulate 786-O cell viability in a dose-dependent manner. In addition, flow cytometry assay revealed that oleic acid delayed 786-O cell apoptosis in a concentration-dependent manner. Following this, specific signaling molecules identified in previous studies to be involved in this mechanism were investigated. Western blot analysis revealed that oleic acid treatment upregulated ILK expression in a concentration-dependent manner. Overexpression of ILK in tumor cells has been found to result in anchorage-independent cell growth, cell cycle progression and tumorigenicity (21). In the current study, overexpression of ILK was found to increase the phosphorylation of Akt on Ser-473, which was consistent with previous observations reporting that the activity of ILK is regulated in a PI3-kinase-dependent manner. In addition, when siRNA was used to target and knock down ILK, the effects of oleic acid on 786-O cells growth were weakened and the expression of ILK was suppressed. These results indicate that oleic acid may regulate RCC cell development through the PI3K/ILK/Akt pathway.

GPR40 is a membrane-bound receptor paired with medium- and long-chain fatty acids as endogenous ligands (22). Briscoe *et al* found that GPR40 was highly expressed in ob/ob mice and may be involved in cell proliferation (23). In the breast cancer cell line, MCF-7, GPR40 was found to be significantly increased at the start and end of cell proliferation and silencing the GPR40 gene using RNA interference was found to suppress oleate-induced cell proliferation (24,25). In the current study, GPR40 was also upregulated by oleic acid treatment and GPR40 was hypothesized to activate the signals associated with cell growth, including ILK and Akt. Akt kinase is activated by phosphorylation at S473 in the regulatory tail by phosphoinositide-dependent kinase, PDK-2, the identity of which is cell or tissue-specific and its activity is highly regulated (26). To date, ∼10 kinases have been demonstrated to function as a PDK-2, including ILK, PKC, PKA and the mTOR complex (27). Consistent with these observations, ILK is activated by GPR40 combined with oleic acid treatment and functions as a PDK-2 to regulate the Akt pathway in RCC.

**Figure 5:**
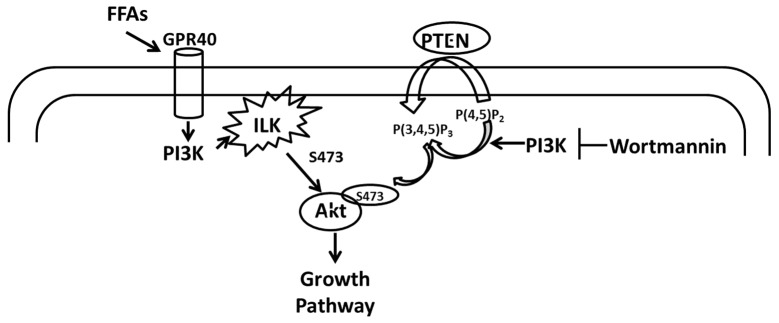
Schematic representation of oleic acid signaling in human renal cell carcinoma (RCC). Oleic acid activates the Akt pathway through stimulation of ILK, identified as one of the kinases with PDK-2 activity in human RCC. The mechanisms involved in this action are not well understood but may be GPR40-dependent. FFA, free fatty acid; GPR40, G protein-coupled receptor 40; ILK, integrin-linked kinase; PTEN, phosphatase and tensin homolog deleted on chromosome 10; P(4,5)P_2_, phosphatidylinositol-4,5-bisphosphate; P(3,4,5)P_3_, phosphatidylinositol-3,4,5-trisphosphate.

In summary, the results of this study indicate the following cascade of events in response to oleic acid in 786-O cells (Fig. 5). Unsaturated FFA binds to GPR40 and may also bind other FFA receptors, resulting in the activation of PI3K, ILK, Akt and subsequent promotion of cell growth. These results provide a novel mechanism for the action of oleic acid in RCC cells on cell growth by demonstrating that this monounsaturated FFA functions as an extracellular signaling molecule to regulate 786-O cell proliferation via the GPR40/ILK/Akt pathway. This pathway may represent a potential therapeutic target and link between insulin resistance, obesity, type 2 diabetes and cancer.

## References

[1] Sourbier C, Massfelder T (2006). Parathyroid hormone-related protein in human renal cell carcinoma. Cancer Lett.

[2] Schrader AJ, Varga Z, Hegele A, Pfoertner S, Olbert P, Hofmann R (2006). Second-line strategies for metastatic renal cell carcinoma: classics and novel approaches. J Cancer Res Clin Oncol.

[3] Tolle A, Jung M, Lein M (2009). Brain-type and liver-type fatty acid-binding proteins: new tumor markers for renal cancer?. BMC Cancer.

[4] Escudier B (2007). Advanced renal cell carcinoma: current and emerging management strategies. Drugs.

[5] Habib SL, Prihoda TJ, Luna M, Werner SA (2012). Diabetes and risk of renal cell carcinoma. J Cancer.

[6] Drabkin HA, Gemmill RM (2010). Obesity, cholesterol and clear-cell renal cell carcinoma (RCC). Adv Cancer Res.

[7] Navarro-Tito N, Soto-Guzman A, Castro-Sanchez L, Martinez-Orozco R, Salazar EP (2010). Oleic acid promotes migration on MDA-MB-231 breast cancer cells through an arachidonic acid-dependent pathway. Int J Biochem Cell Biol.

[8] Suburu J, Chen YQ (2012). Lipids and prostate cancer. Prostaglandins Other Lipid Mediat.

[9] Yun MR, Lee JY, Park HS (2006). Oleic acid enhances vascular smooth muscle cell proliferation via phosphatidylinositol 3-kinase/Akt signaling pathway. Pharmacol Res.

[10] Vinciguerra M, Carrozzino F, Peyrou M (2009). Unsaturated fatty acids promote hepatoma proliferation and progression through downregulation of the tumor suppressor PTEN. J Hepatol.

[11] Hannigan G, Troussard AA, Dedhar S (2005). Integrin-linked kinase: a cancer therapeutic target unique among its ILK. Nat Rev Cancer.

[12] McDonald PC, Oloumi A, Mills J (2008). Rictor and integrin-linked kinase interact and regulate Akt phosphorylation and cancer cell survival. Cancer Res.

[13] Soto-Guzman A, Navarro-Tito N, Castro-Sanchez L, Martinez-Orozco R, Salazar EP (2010). Oleic acid promotes MMP-9 secretion and invasion in breast cancer cells. Clin Exp Metastasis.

[14] Stemmer K, Perez-Tilve D, Ananthakrishnan G (2012). High-fat-diet-induced obesity causes an inflammatory and tumor-promoting microenvironment in the rat kidney. Dis Model Mech.

[15] Bartsch H, Nair J, Owen RW (1999). Dietary polyunsaturated fatty acids and cancers of the breast and colorectum: emerging evidence for their role as risk modifiers. Carcinogenesis.

[16] Willett WC (1997). Specific fatty acids and risks of breast and prostate cancer: dietary intake. Am J Clin Nutr.

[17] Han S, Sun X, Ritzenthaler JD, Roman J (2009). Fish oil inhibits human lung carcinoma cell growth by suppressing integrin-linked kinase. Mol Cancer Res.

[18] Hammamieh R, Chakraborty N, Miller SA (2007). Differential effects of omega-3 and omega-6 Fatty acids on gene expression in breast cancer cells. Breast Cancer Res Treat.

[19] Soto-Guzman A, Robledo T, Lopez-Perez M, Salazar EP (2008). Oleic acid induces ERK1/2 activation and AP-1 DNA binding activity through a mechanism involving Src kinase and EGFR transactivation in breast cancer cells. Mol Cell Endocrinol.

[20] Gorjao R, Hirabara SM, de Lima TM, Cury-Boaventura MF, Curi R (2007). Regulation of interleukin-2 signaling by fatty acids in human lymphocytes. J Lipid Res.

[21] Persad S, Dedhar S (2003). The role of integrin-linked kinase (ILK) in cancer progression. Cancer Metastasis Rev.

[22] Del GS, Bugliani M, D’Aleo V (2010). G-protein-coupled receptor 40 (GPR40) expression and its regulation in human pancreatic islets: the role of type 2 diabetes and fatty acids. Nutr Metab Cardiovasc Dis.

[23] Briscoe CP, Tadayyon M, Andrews JL (2003). The orphan G protein-coupled receptor GPR40 is activated by medium and long chain fatty acids. J Biol Chem.

[24] Yonezawa T, Katoh K, Obara Y (2004). Existence of GPR40 functioning in a human breast cancer cell line, MCF-7. Biochem Biophys Res Commun.

[25] Hardy S, St-Onge GG, Joly E, Langelier Y, Prentki M (2005). Oleate promotes the proliferation of breast cancer cells via the G protein-coupled receptor GPR40. J Biol Chem.

[26] Hanada M, Feng J, Hemmings BA (2004). Structure, regulation and function of PKB/AKT - a major therapeutic target. Biochim Biophys Acta.

[27] Dong LQ, Liu F (2005). PDK2: the missing piece in the receptor tyrosine kinase signaling pathway puzzle. Am J Physiol Endocrinol Metab.

